# Au–Si plasmonic platforms: synthesis, structure and FDTD simulations

**DOI:** 10.3762/bjnano.9.241

**Published:** 2018-09-28

**Authors:** Anna Gapska, Marcin Łapiński, Paweł Syty, Wojciech Sadowski, Józef Eugeniusz Sienkiewicz, Barbara Kościelska

**Affiliations:** 1 Faculty of Applied Physics and Mathematics, Department of Solid State Physics, Gdansk University of Technology, Gabriela Narutowicza 11/12, 80-233 Gdansk, Poland; 2 Faculty of Applied Physics and Mathematics, Department of Theoretical Physics and Quantum Information, Gdansk University of Technology, Gabriela Narutowicza 11/12, 80-233 Gdansk, Poland

**Keywords:** Au plasmonic platforms, dewetting, eutectic, finite-difference time domain (FDTD)

## Abstract

Plasmonic platforms based on Au nanostructures have been successfully synthesized by directional solidification of a eutectic from Au and the substrate. In order to determine homogeneous shape and space distribution, the influence of annealing conditions and the initial thickness of the Au film on the nanostructures was analyzed. For the surface morphology studies, SEM and AFM measurements were performed. The structure of platforms was investigated using XRD and XPS methods. Structural investigations confirmed, that nanostructures consist of metallic Au, growing along the [111] direction. The most homogeneous seems to be the platform obtained by solidification of a 2.8 nm Au film, annealed at 550 °C for 15 min. This sample was subsequently chosen for theoretical calculations. Simulations of electromagnetic field propagation through the produced samples were performed using the finite-difference time domain (FDTD) method. The calculated absorbance, as a result of the FDTD simulation shows a quite good agreement with experimental data obtained in the UV–vis range.

## Introduction

The evolution of metal thin films into nanostructures under various thermal conditions has been repeatedly studied for many years. However, the number of works on this subject has increased significantly over the last ten years. This is mainly due to technological needs related to the development of optoelectronics, photonics, electronics and energy conversion systems, fields in which metallic nanostructures found their application. Of course, this increase was closely related to the development of novel manufacturing technologies. One of the most interesting fields based on metal nanostructures is plasmonics. The use of plasmonic effects opens up many interesting possibilities, as for example photoluminescence intensification or subwavelength propagation. There are a lot of methods for the manufacturing of plasmonic platforms. Some of them are relatively simple and cost-effective, but sizes and shapes of the obtained metallic nanoparticles are not homogeneous. On the other hand, there are also methods that can yield more homogeneous distributions of nanostructures, but are far more complex and expensive, such as electron beam lithography. One promising technique that could be used to fabrication of plasmonic platforms is the method based on the directional solidification of eutectics. In this process, two or more phases can grow, depending on the phase equilibrium system. The formation of various geometries during eutectic solidification is also possible. In addition, the existence of a eutectic between the substrate, to which a metal in the form of a thin layer is applied, and the metal, significantly reduces the melting point of the metal. This is clearly visible in the case of the Au–Si eutectic, which has a melting temperature of ca. 363 °C [1–4]. It seems that in the case of directional solidification of eutectics as a method of manufacturing of nanostructures, the platforms are homogeneous. Unfortunately, as it can be found in literature, that eutectic-based growth may lead to an increase of inclusions of another phase in metallic structures [2,4–6]. When nanostructures are formed from thin metal films, directional eutectic growth is not the only process that needs to be considered. As-deposited thin films are most often metastable and can dewet or agglomerate with increase of temperature. During annealing of thin films, small fluctuations of the film thickness appear leading to the creation of voids in the film and their subsequent growth. This can happen well below melting temperature of the film so dewetting occurs while the film remains in the solid state. When the temperature of the system is increased up to the eutectic temperature, the solid–liquid transition takes place and liquid droplets remain on the support surface [2]. In plasmonics, one of the most commonly used metal is Au, characterized by a large number of free electrons, which leads to a high plasma frequency and a negative real permittivity over a wide range of frequencies. Taking into account the existence of a eutectic from Au and Si, which gives the possibility of producing metal nanostructures on Si, and the aforementioned use of Au in plasmonic systems, it seems that plasmonic platforms based on Au nanostructures produced by directional solidification of the eutectic would be suitable for photonics applications. In the design of this type of arrangements, it is particularly important to know the spatial field distributions, which could be helpful in identifying some structures in the investigated system (so called hot spots), responsible for light enhancement at some frequencies. This can be achieved by using numerical simulations. The method often used for this type of calculation is the finite-difference time domain (FDTD) method. The method allows one to find the spatial distributions of all components of an electromagnetic field propagating through the investigated system, at selected time intervals. Applying in the next step the discrete Fourier transform (DFT) leads to a change from the time domain to the frequency domain, which in turn gives results of the properties in a wide-range frequency spectrum, e.g., absorbance and transmittance of the sample, which could be directly compared to results from UV–vis measurements. The combination of experimental and computational methods in the design of materials or systems offers a great possibility to develope the most desirable parameters.

This paper presents the results of manufacturing Au-based plasmonic platforms by directional solidification of a eutectic and the study of their structure compared with the results obtained by calculations by FDTD method.

## Experimental

**Figure 1:**
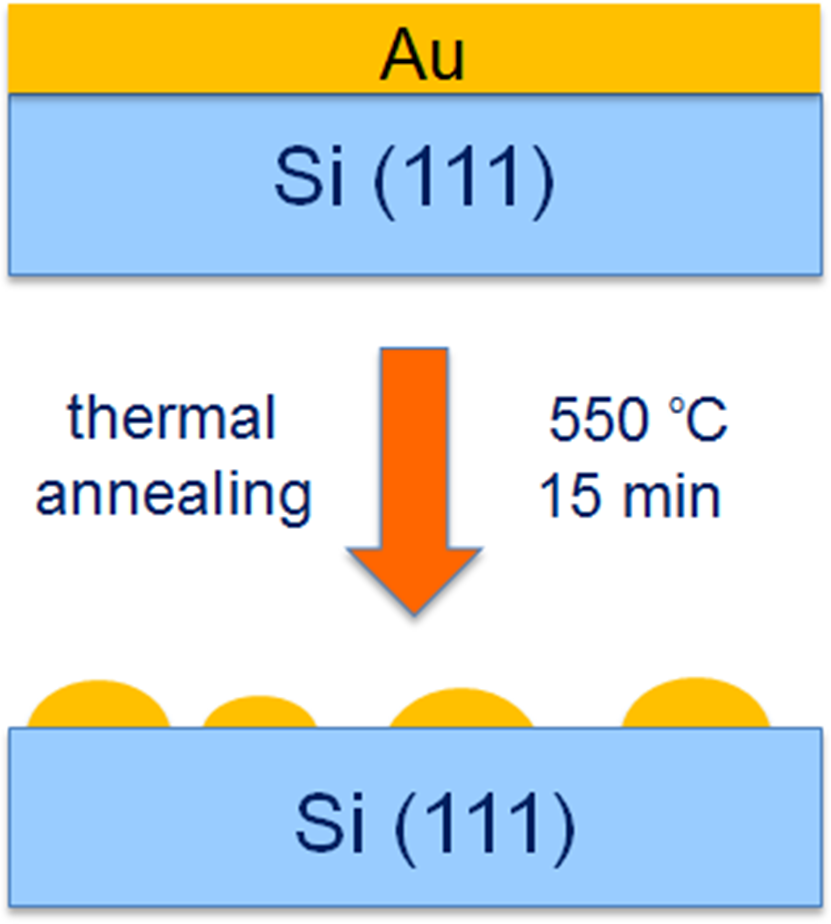
Scheme of the formation of the gold nanoislands.

Au nanostructures were prepared on Si(111) as a substrate. The substrates (1 × 1 cm^2^ of area) were cleaned with acetylacetone and then rinsed in ethanol. Thin Au films (with thicknesses in a range of 1.7–5.0 nm) were deposited using a table-top dc magnetron sputtering coater (EM SCD 500, Leica) under pure Ar plasma conditions (Argon, Air Products 99.999%). The Au target had 99.99% purity, the rate of Au layer deposition was about 0.4 nm·s^−1^ and the incident power was 32 W. The thickness of the films was measured in situ by a quartz crystal microbalance. The films were subsequently annealed at different temperatures (up to 600 °C) in air in a hot furnace. A scheme of the formation of the gold nanostructures is shown in Figure 1.

To analyze the surface morphology of the samples, a FEI Quanta FEG 250 scanning electron microscope (SEM) operated at 10 kV and an atomic force microscope (AFM) Omicron UHV VT SPM XA were used. AFM images were recorded in contact mode using a Si tip with a radius lower than 10 nm. The structure of the samples was examined by using a Phipips X’Pert diffractometer, using Cu Ka radiation in a 2θ range of 10–70° with a step size of 0.006° at 1 s per step. The quality of the obtained nanostructures and valence states of Au was measured using X-ray photoelectron spectroscopy (XPS) with an Omicron NanoTechnology spectrometer with 128-channel collector. XPS measurements were performed at room temperature under ultra-high vacuum conditions, below 1.1 × 10^−8^ mbar. The photoelectrons were excited by an Mg Kα X-ray source. The X-ray anode was operated at 15 keV and 300 W. An Omicron Argus hemispherical electron analyzer with a round aperture of 4 mm was used for analyzing the emitted photoelectrons. The binding energies were corrected using the background C 1s line (285.0 eV). XPS spectra were analyzed with the Casa-XPS software using a Shirley background subtraction and Gaussian–Lorentzian curves for fitting. UV–vis spectra were recorded by a double-beam Thermo Fisher Scientific Evolution 220 spectrophotometer in reflectance mode. The spectra were recorded in a range of 200–1100 nm.

Simulations of electromagnetic field propagation through the produced samples were performed using FDTD calculations [7–8]. The method allows one to find the spatial distribution of all components of electromagnetic field propagating through the investigated system, at selected time intervals. Maxwell equations are solved numerically on the discrete grid, the so-called Yee grid [9]. For the FDTD calculations, the commercial software Omnisim/CrystalWave (https://www.photond.com/products/omnisim.htm by Photon Design, Oxford, UK) was used to simulate the experimental conditions, as well as to analyze the experimental data. The flexible interface allowed for a quite convenient modeling of the studied system. Here, in order to obtain the actual arrangement and size of nanoislands, a supporting software has been written that reads the SEM image, finds contours of nanoislands and transform them to the proper OmniSim input. As a result, the performed FDTD calculations were based on the precisely experimental formation of gold nanostructures.

Nevertheless, it has to be noted, that the Maxwell’s FDTD method is able to give only quantitative results for electromagnetic field distribution on the nanoparticle plates. One of the ways to go beyond this classical model is to add to the Maxwell’s FDTD method the quantum-mechanical description of the near-field distribution by using the time-dependent Schrödinger equations [10]. However, here one of the difficulties lies in the requirement for very small time steps.

## Results and Discussion

### Structure and UV–vis absorption

**Figure 2:**
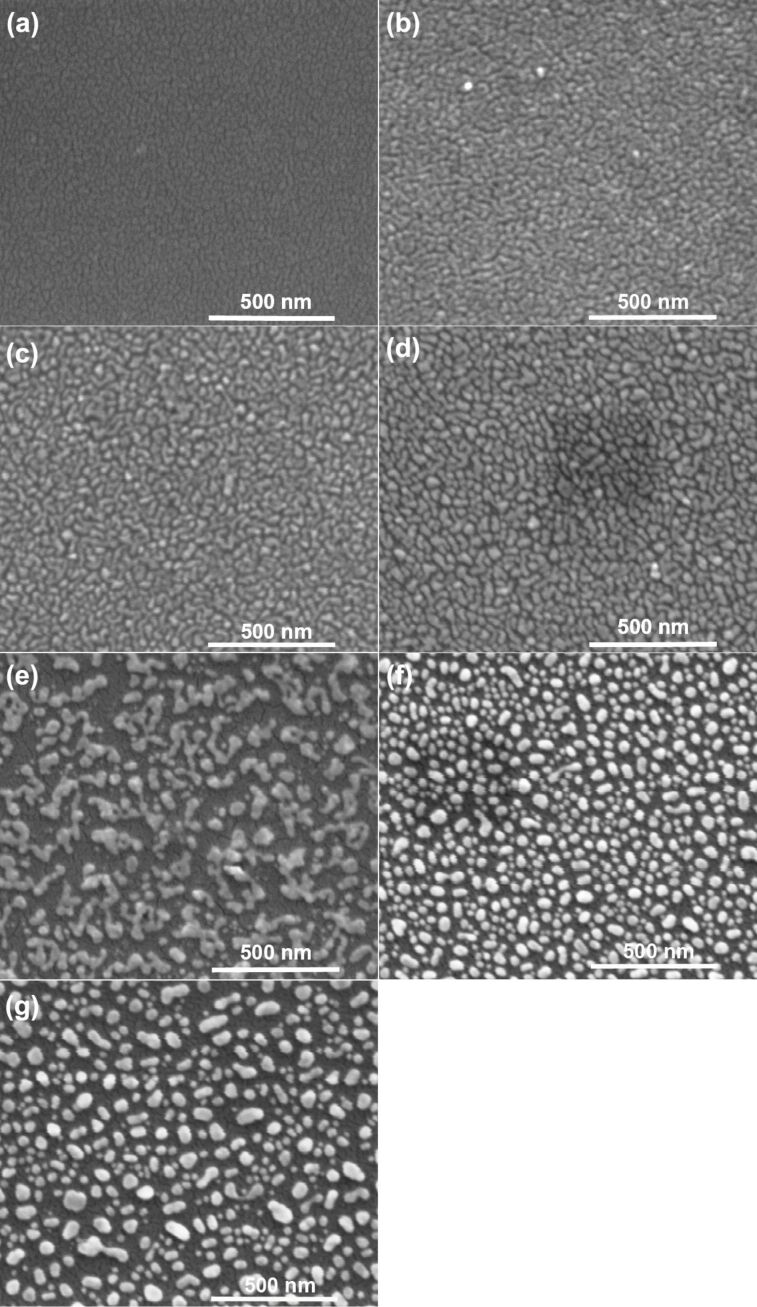
SEM images of 2.8 nm Au films on a Si(111) substrate: a) as prepared, annealed at b) 150 °C, c) 200 °C, d) 250 °C, e) 300 °C, f) 350 °C, g) 363 °C for 15 min.

The formation of nanostructures from 2.8 nm thick Au thin films, at different annealing temperatures is shown in Figure 2b–g. All films presented in these pictures were annealed for 15 min. For comparison, an SEM image of the as-prepared film is shown in Figure 2a. The surface of the as-deposited film is not smooth. Some roughness can be noticed, which increases during annealing. It can be seen in Figure 2b that annealing already at 150 °C caused the appearance of voids in the film, which can be a consequence of atom fluctuations. At 200 °C, these fluctuations are large enough to create grooves reaching the substrate surface (Figure 2c). The situation is similar for the film annealed at 250 °C (Figure 2d). At 300 °C (Figure 2e), islands of very irregular shape are visible on the surface. More regular islands are seen after thermal annealing at 350 °C (Figure 2f). It is clear that the formation of gold nanostructures on silicon starts below the eutectic temperature where the main force leading to the nanostructures formation is the reduction of the surface energy. This behavior of thin films can be explained through dewetting. Dewetting of metallic thin and ultra-thin films, although known and widely studied for many years, it is still an interesting phenomenon [11–18]. Dewetting of films is driven by two main processes. The first of them is based on nucleation and growth of holes. The growth of holes occurs through the gathering of material from which the layer is made, along the perimeter of the hole, which results in a raised rim around the hole. Nucleation can occur in two ways, as homogeneous nucleation, when holes are caused by small thermal-density fluctuations (homogeneous dewetting), or as heterogeneous nucleation, which is caused by defects in the layer or on the interface of the layer and the substrate (heterogeneous dewetting). In the second process, called spinodal dewetting, instability of the film against thermally activated surface waves leads to spontaneous rupturing of the film. Generally speaking, dewetting is a complex phenomenon and it is difficult to judge from the experimental data alone which of the abovementioned processes occurs in the case of thin metallic layers. Often the formation of nanostructures from thin metal layers was explained based only on spinodal dewetting [13,15–18]. Although these studies concern films with a thickness of more than 10 nm (which is much thicker than the films discussed here) and films are formed on inert substrates, it seems to be possible (based on the above SEM images) that spinodal dewetting takes place also in our samples. On the other hand, many defects are already created when a thin film of metal is deposited on the Si(111) surface, so heterogeneous dewetting must be also considered.

During the formation of nanostructures from thin metallic films, it is necessary to take into account, in addition to dewetting, also directional solidification of eutectics [3,5,19–21]. In the gold–silicon system, an Au-rich eutectic melts at a temperature of 363 °C. At this temperature, because the solubility of Au in Si is negligible, the Au–Si eutectic melt does not wet the Si surface [3]. The value of the contact angle in this system is about 40° [2]. As a consequence, Au–Si nanodroplets do not dissolve on the Si surface, forming the nanostructures during cooling. In the case of the studied layers, it cannot be unequivocally determined which of the two processes discussed above dominates at the eutectic temperature. Especially because the layers are very thin and do not form a perfectly uniform surface after their deposition. A SEM image of the film annealed at the eutectic temperature is presented in Figure 2g. The nanoparticles formed at this temperature are not spherical and their distribution is not homogeneous.

**Figure 3:**
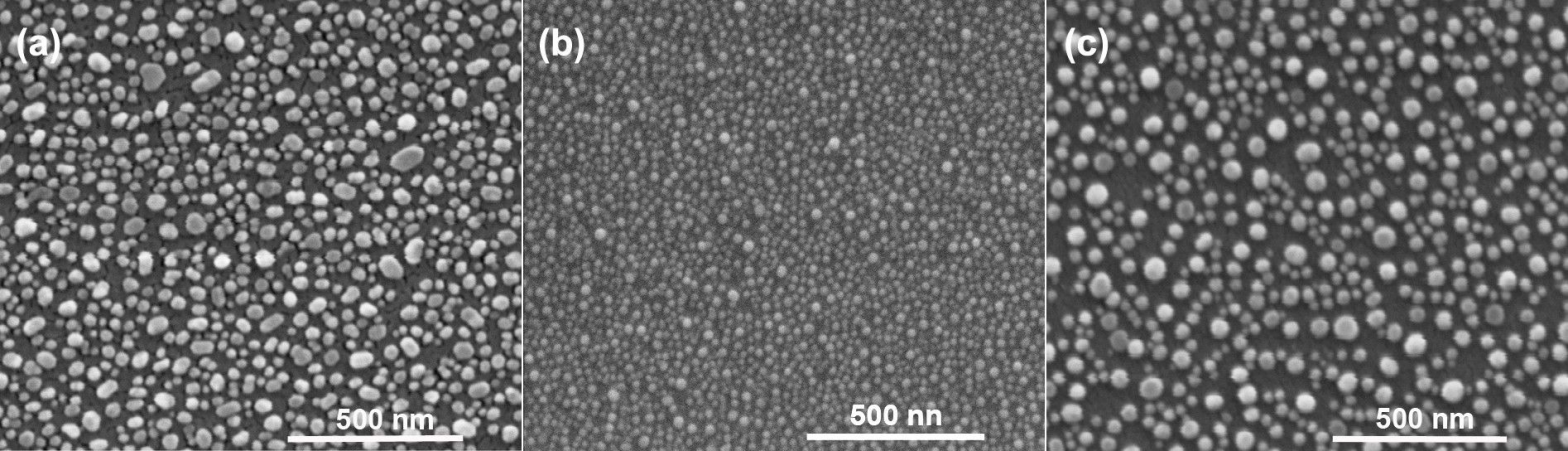
SEM images of 2.8 nm Au films on a Si(111) substrate annealed at a) 450 °C, b) 550 °C, c) 600 °C for 15 min.

**Figure 4:**
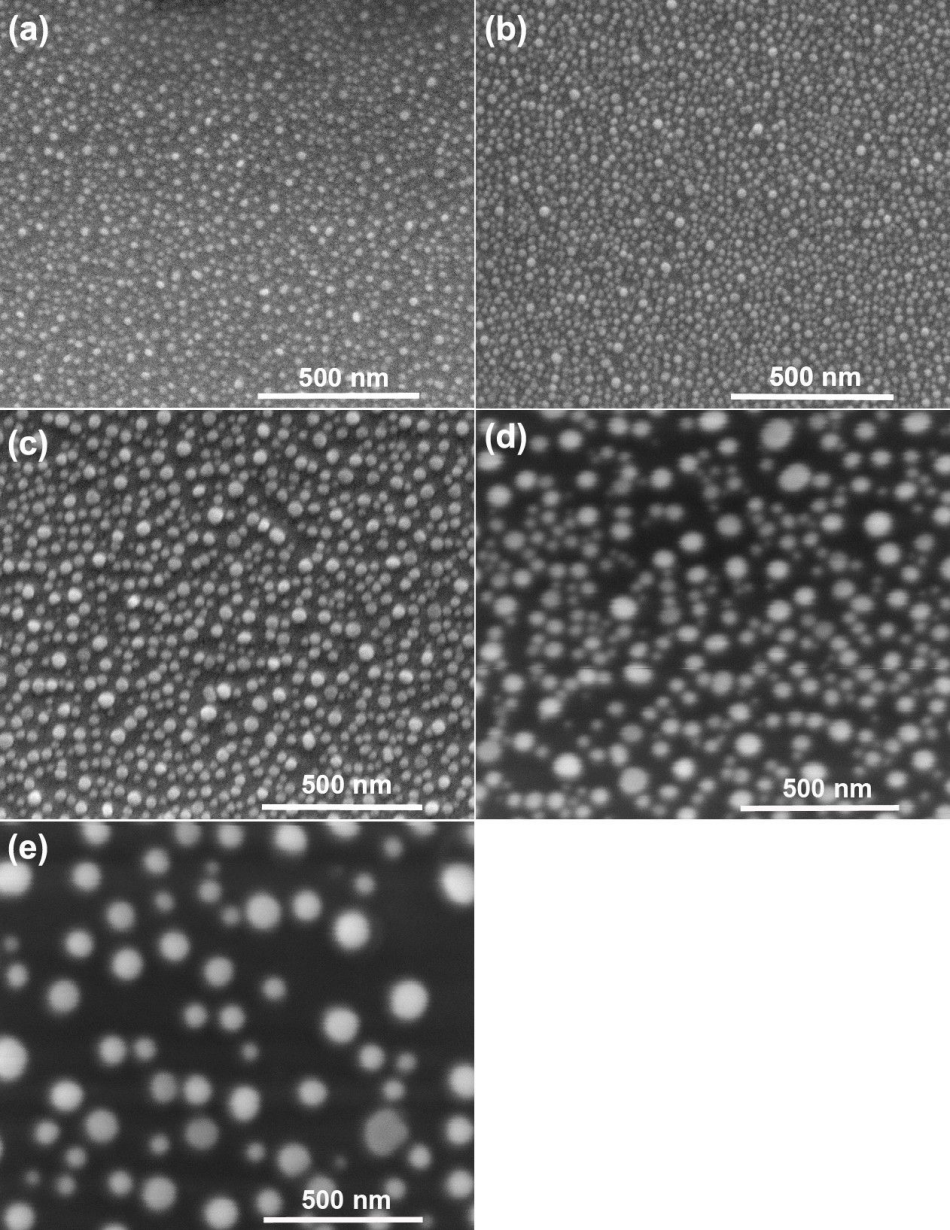
SEM images of Au films of different thicknesses on a Si(111) substrate annealed at 550 °C for 15 min: a) 1.7 nm, b) 2.8 nm, c) 3.1 nm d) 4.0 nm, e) 5.0 nm.

**Figure 5:**
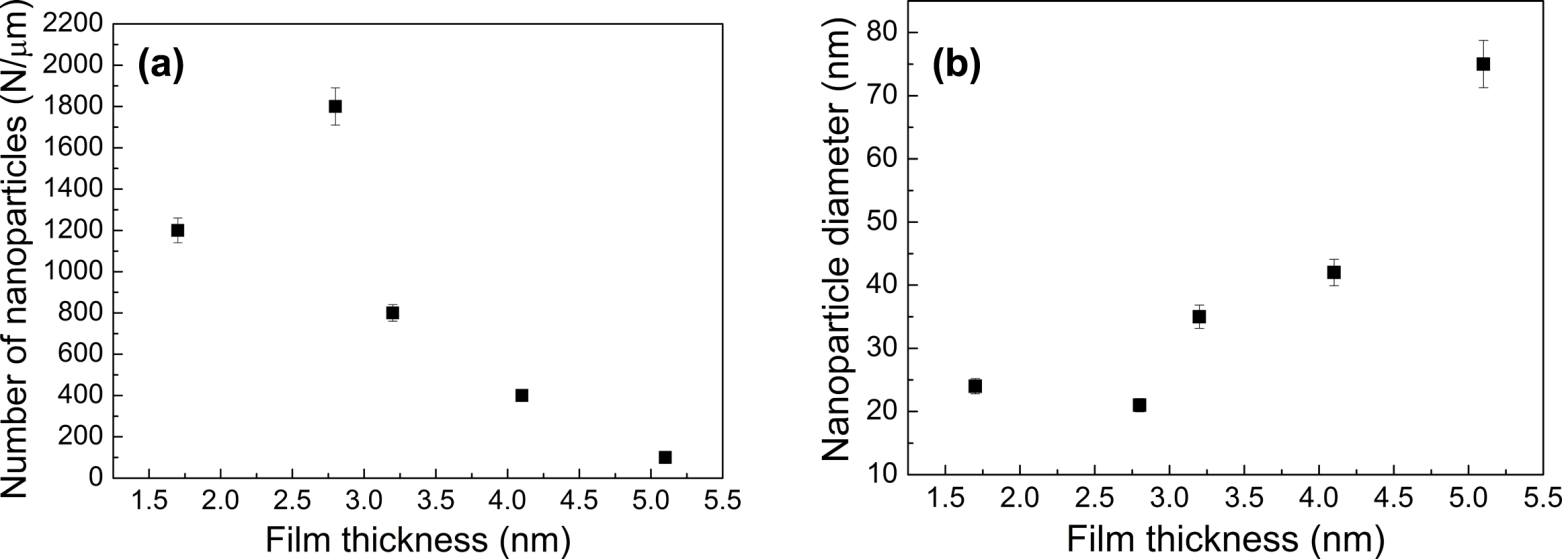
a) Number of nanoparticles and b) average diameter of nanoparticles as function of the Au film thickness on Si(111) substrates annealed at 550 °C for 15 min. Errors are below 5%.

To study the influence of temperature on size and distribution of nanoparticles above the eutectic temperature, films were annealed up to 600 °C. Exemplary images of the 2.8 nm thick film are shown in Figure 3. After annealing at 450 °C (Figure 3a), nanoparticles are still not spherical. The shape of nanoparticles changes with increasing temperature, and at 550 °C the nanoparticles seem to be almost spherical and uniformly distributed on the surface (Figure 3b). After heating the layer at 600 °C, the average size of the nanoparticles increased (Figure 3c). An annealing temperature of 550 °C was subsequently chosen to observe the formation of nanoparticles from layers of different thicknesses of 1.7, 2.8, 3.1, 4.0 and 5.0 nm. SEM images of these samples annealed at 550 °C for 15 min are presented in Figure 4. When the thickness of the films increases, the average diameter of nanoparticles also increases, but the amount of nanoparticles decreases (Figure 5). This behavior is consistent with the literature, but it can be also found, that during the annealing of polycrystalline films, an increase in grain size takes place until they reach a size approximately equal to twice the initial film thickness [11]. However, these samples were thicker than our layers and, such effects were not observed in our samples. Based on the relation describing the mean nanoparticle diameter (*D*) and the mean spacing between them (*s*) with the initial film thickness (*h*), it is possible to specify the type of dewettting. As it was shown in literature, for spinodal dewetting these dependences are *D* 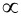 *h*^5/3^ and *s* 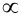 *h*^2^ [13,18]. Unfortunately, on the basis of Figure 5b, due to the small number of data points, we are not able to determine such dependencies. So our considerations are therefore only qualitative.

**Figure 6:**
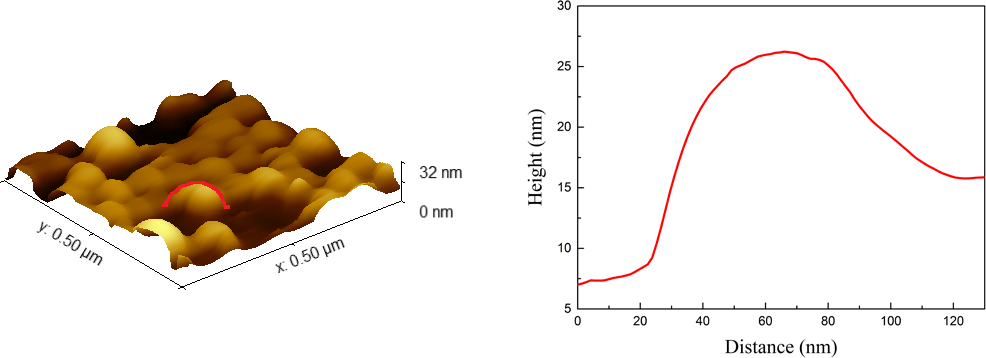
Left: AFM image of a 2.8 nm Au film on a Si(111) substrate annealed at 550 °C for 15 min; right: cross section of a selected nanoisland.

The most uniform nanostructures, both in terms of size and distribution on the surface, occur in the sample obtained from a layer of 2.8 nm thickness annealed at 550 °C for 15 min. AFM image and cross section of this sample are shown in Figure 6. Based on the AFM image, the RMS value (root mean square) of the film was calculated to be 5.7 nm. The grains are slightly oval, which was not shown in the SEM pictures. From the cross section it follows that the height of the nanoparticles is smaller than the horizontal width.

**Figure 7:**
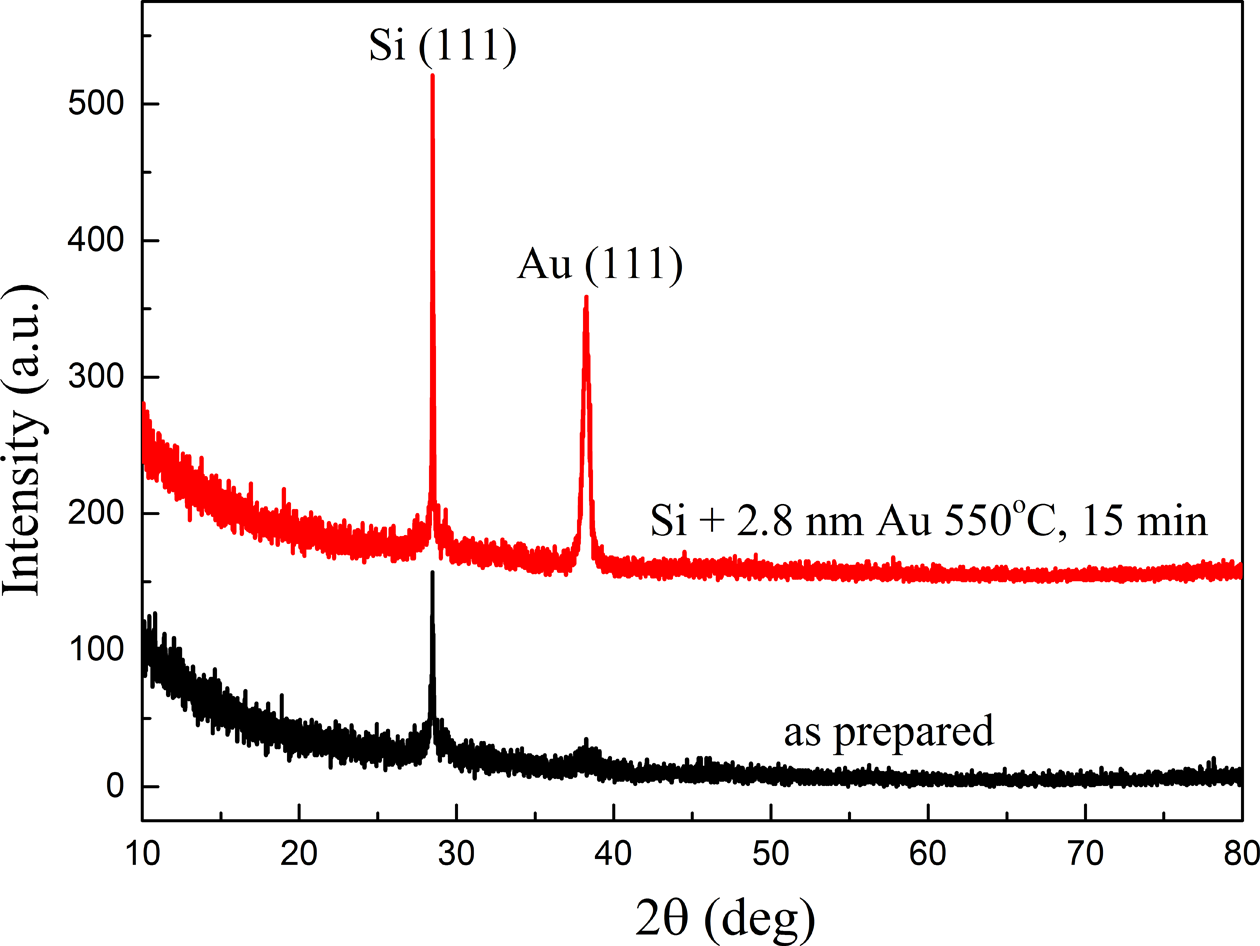
XRD result of a 2.8 nm Au film annealed at 550 °C for 15 min and an as-prepared 2.8 nm Au film on Si(111) substrates.

The crystalline structure of as-deposited layers and nanoparticles formed during their heating was examined by using XRD. Exemplary results for the as-prepared 2.8 nm film and the nanostructures after annealing at 550 °C for 15 min are presented in Figure 7. Only two fairly wide peaks of low intensity are seen in the diffraction pattern of the as-prepared film. The first one, at an angle of about 28.7° corresponds to the (111) crystal plane of silicon used as substrate. The second, at an angle of about 38.2° comes from the (111) plane of Au. The diffractogram of the sample after annealing does not differ very much. The peak corresponding to the (111) plane of Si is more intense than in the case of the as-prepared layer, probably because after the formation of nanostructures, the large part of Si support is exposed. In the case of Au, still only one peak from (111) crystal plane is visible in the diffractogram, but with a higher intensity than in the as-prepared sample. However the size of the nanostructures that formed after annealing significantly exceeds the initial thickness of the layer (Figure 6), so the intensity of peaks must be higher. It seems that the Au layer grows in the [111] direction, but with such a low-peak intensity it is difficult to say this unambiguously, as the remaining peaks coming from other Au crystal planes, equal to at most half the height of the (111) peak, may not be visible. The directional growth of the layer and later directional growth of the nanostructures, leads to the existence of stress in the layer because the lattice constants between them can be different. In turn, this can be a driving force for the dewetting process [15,22].

**Figure 8:**
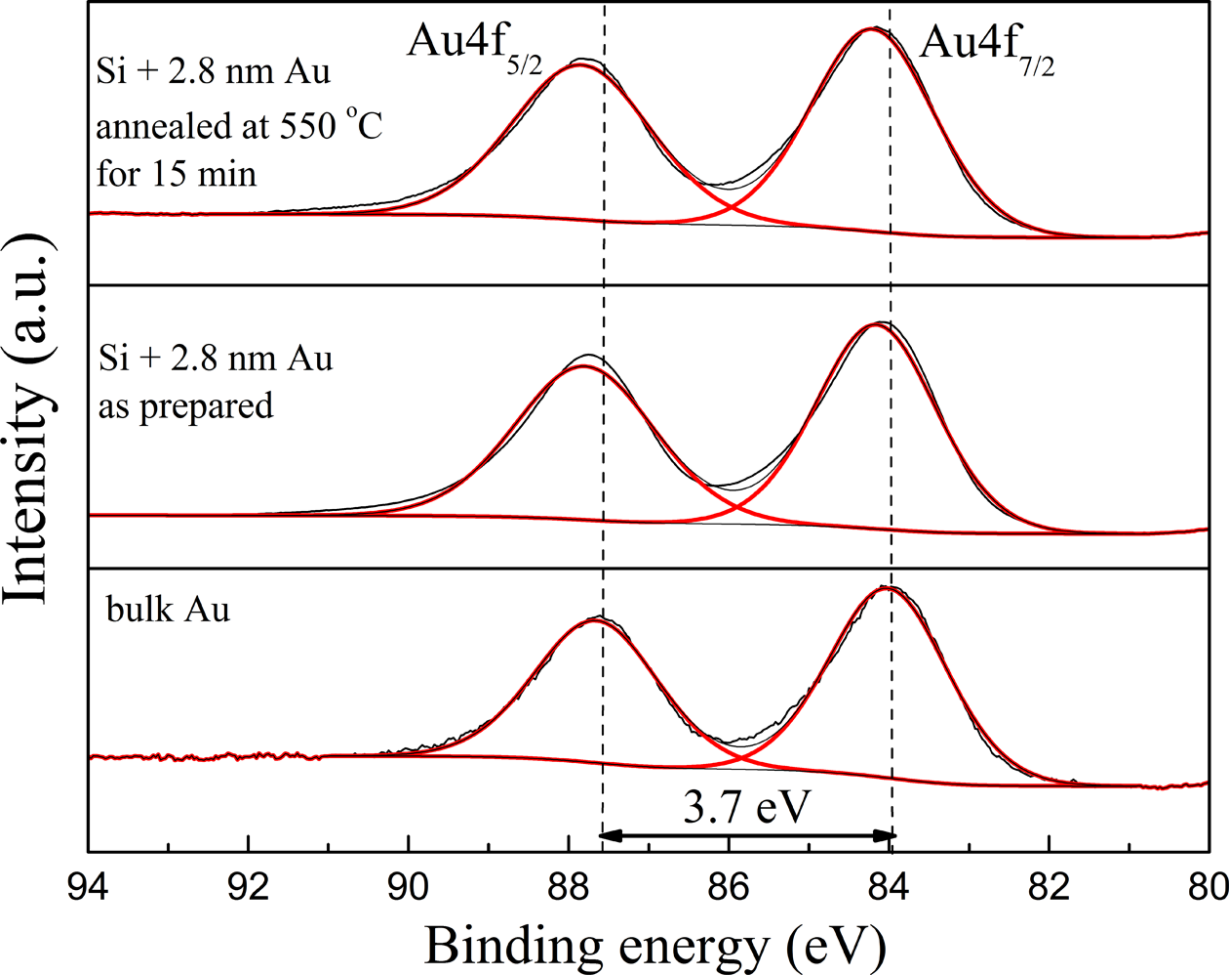
XPS (Au region) results for the 2.8 nm Au thin film on a Si(111) substrate annealed at 550 °C for 15 min, the as-prepared 2.8 nm Au film on Si(111) and bulk Au.

**Figure 9:**
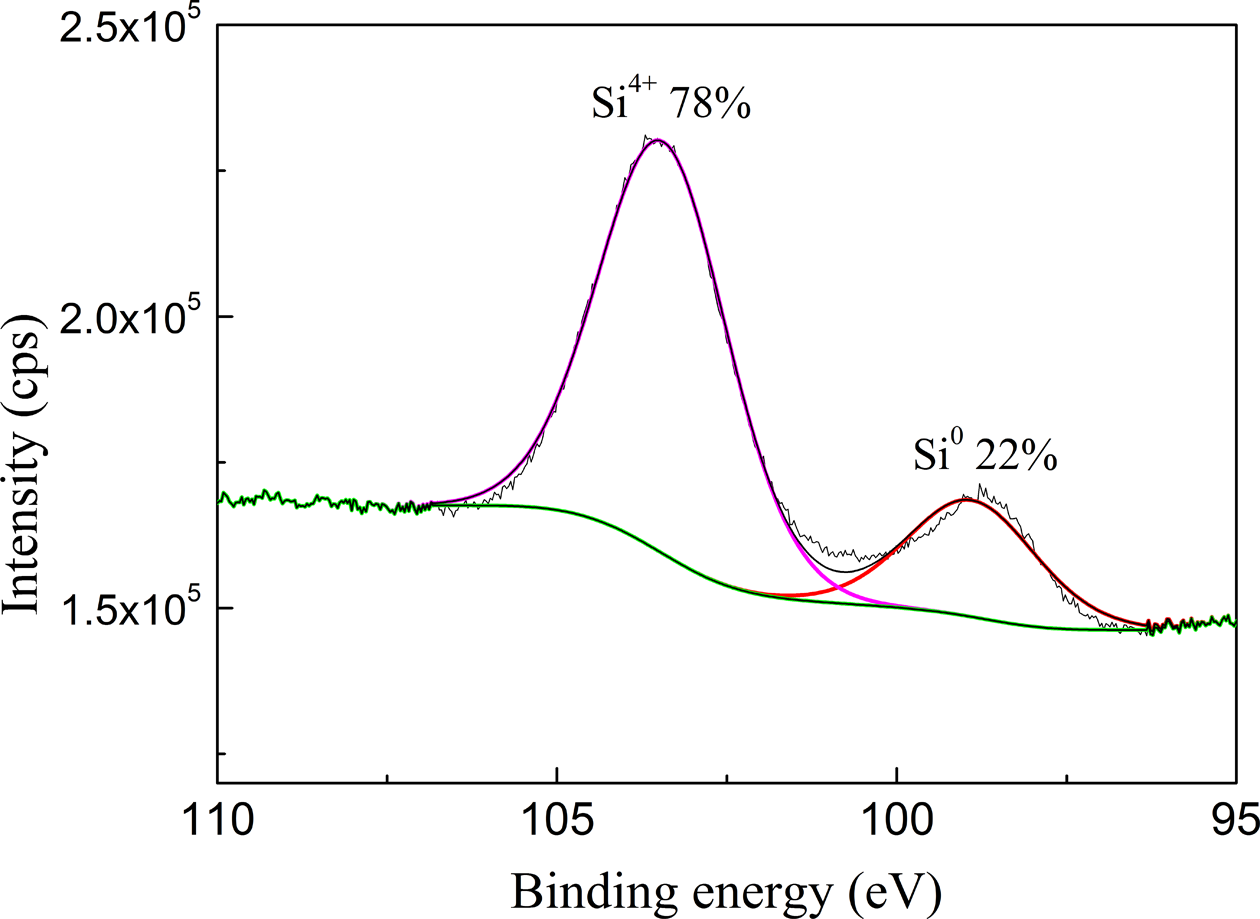
XPS result (Si region) for the 2.8 nm Au film on a Si(111) substrate annealed at 550 °C for 15 min.

XPS was used to determine the valence state of Au films and Au nanostructures. The Au 4f doublet of the 2.8 nm thin film after annealing at 550 °C for 15 min is presented in Figure 8. For comparison the same spectra measured for the as-prepared film and for bulk Au are added. The binding energy of the Au 4f core-level shifts, depending on cluster size, cluster–substrate interactions, cluster morphology and charge transfer between cluster and substrates [23–24]. Additionally, the peak ratio of the Au 4f_7/2_ and 4f_5/2_ excitations differs from the statistical ratio of 4:3 and the linewidths (FWHM) are not equal. The size of the clusters can also affect the symmetry of the Au 4f_7/2_ peak on the side of higher binding energies, and the asymmetry increases with decreasing cluster size. In our nanostructures the shift of binding energy is also observed. Also a small asymmetry of the 4f_7/2_ peak can be noticed on its higher-energy side. However there is no difference between the spectra of nanostructures and thin film. In the XPS results only the states corresponding to metallic gold are observed. The nanostructures presented in Figure 8 were annealed at a temperature much higher than the eutectic temperature, but one cannot see the Au_100−_*_x_*Si*_x_* intermetallic alloy, which should be observed for the samples manufactured through directional solidification of the eutectic. However, the occurrence of Au–Si inclusions is not a rule [19]. Au–Si bonds were neither observed in the energy range corresponding to the Si region, shown in Figure 9 for the 2.8 nm thin film annealed at 550 °C for 15 min. The peak at the energy position of 98.9 eV may be associated to metallic Si whereas the higher-energy peak, at 103.5 eV, is close to SiO_2_. The second peak can be explained by presence of very thin film of native SiO_2_ formed on Si substrates in air. During the heating of Au films on Si at temperatures above the eutectic temperature, an intermetallic thin layer of Au–Si is formed on the contact interface. However, it is usually a monoatomic layer that could not be observed with XPS [2,25]. The formation of such a layer would be impeded by the SiO_2_ film formed on the Si surface, which would also explain the absence of Au–Si bonds. On the other hand, research results show that the presence of SiO_2_ is not an efficient diffusion barrier against metal atoms [24], so Au–Si monoatomic layer could be present on the surface of the support.

**Figure 10:**
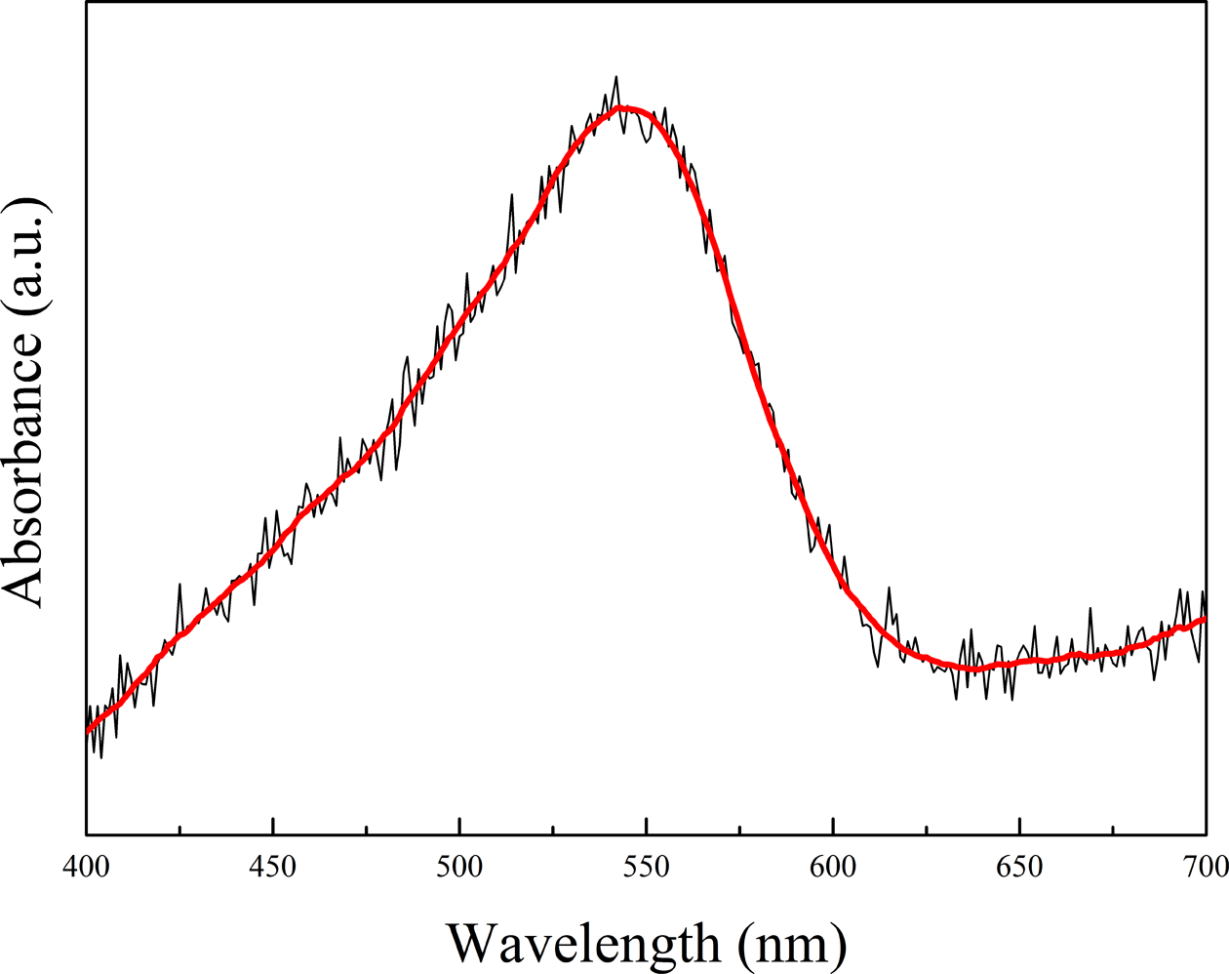
Absorbance spectra recorded for a 2.8 nm Au film on a Si(111) substrate annealed at 550 °C for 15 min.

Exemplary absorbance spectra recorded for the 2.8 nm Au film after annealing at 550 °C for 15 min is presented in Figure 10. A strong maximum corresponding to plasmon resonance is observed at about 545 nm. This sample was subsequently chosen for theoretical calculations.

### FDTD simulation

**Figure 11:**
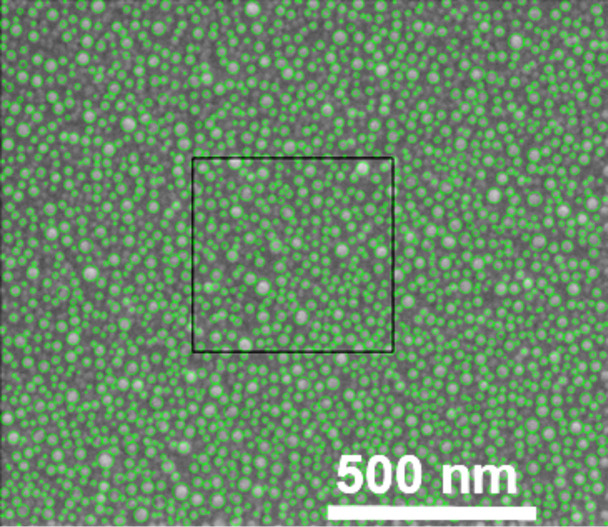
SEM image of the gold nanoislands on the silicon substrate (sample 2.8 nm Au film on Si(111) substrate after annealing at 550 °C for 15 min). Green circles indicate the recognized structures. A sample of 0.5 μm × 0.5 μm size (marked by the black frame) was used for the simulations.

In order to perform the fully three-dimensional FDTD (finite-differences time-domain) simulations, we reproduced the morphological structure of the gold nanoislands on the silicon surface. A sample of 0.5 μm × 0.5 μm size was chosen (Figure 11). It contained 270 nanoparticles that were modeled as full spheres for simplicity. The minimal, maximal and average diameters of the spheres were 3.4, 40.8 and 16.42 nm, respectively. In the simulation, the samples were excited by perpendicularly polarized light waves, then the results were averaged to obtain the non-polarized wave for which all results are presented. While solving the Maxwell equations through FDTD calculations, the complex dielectric function plays a crucial role as it describes dispersion in both metal and dielectric layers in the simulated systems. For metals in an oscillating external field, this function depends on the oscillation frequency, and its proper modeling is a very important step in the simulation process. In our case, the dielectric functions of gold [26] and silicon [27] were fitted to the Lorentz model [28] in the wavelength range of 300–1200 nm. The fitting error was below 5% for Au and below 3% for Si, for both real and imaginary parts.

**Figure 12:**
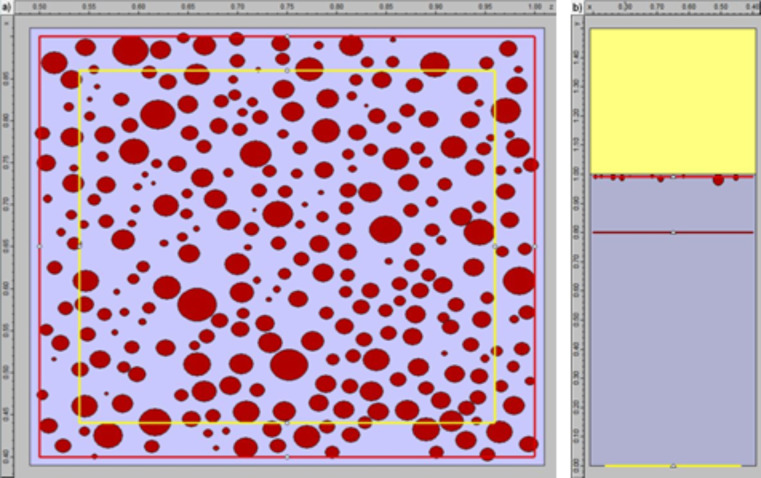
Setup for the FDTD and FDTD/DFT simulations. Left: view from the bottom, *zx*-plane; yellow rectangle – outer border of the light source; red rectangle – outer border of the sensors; brown circles – gold nanoparticles. Right: Side view, *xy*-plane; vellow line – light source, wave propagation in upward direction; red and brown lines – sensors for FDTD and FDTD/DFT simulation sets, respectively; light-blue area – air; yellow area – silicon; brown circles – gold nanoparticles.

The scheme of the system for numerical simulations that imitated the experimental setup is shown in the Figure 12. Two independent simulations were performed, each for different properties of the time envelope of the incident pulse, and different positions of the sensors (virtual, rectangular devices recording flux of the electromagnetic field through their surfaces). In the first simulation, the FDTD method was used for determining the electromagnetic field distribution on the sample. The time envelope of the pulse was set to be rectangular. It provided the single wavelength of 395 nm (which is the off-resonant transition for Si) for exactly 10 fs. The sensor was placed in the *zx*-plane, 10 nm above the Si substrate. The second simulation, by using FDTD and discrete Fourier transform (DFT) analysis, allowed for obtaining the absorbance of the sample in the frequency domain. To switch from the time domain to the frequency domain, FDTD simulations and subsequent discrete Fourier transform were performed. This will be called FDTD/DFT further in the text. The same result could be achieved directly by using the finite-difference frequency-domain (FDFD) method, but this is implemented in OmniSim only for two dimensions. The time envelope of the incident pulse was modeled as bandwidth-limited pulse in the 300–1000 nm range, which corresponds to a 2.5 fs sinusoidal pulse. The sensor was placed in the *zx*-plane, between the light source and the nanoparticle layer, to allow for recording both incident and reflected fluxes. In both cases the pulse propagated along the *y*-axis, perpendicular to the sample surface, and was of a collimated phase front with a power of 1 W; the overall simulation time was set to 40 fs, and the grid size was 1 nm (which is less than a third of the smallest nanoparticle in the structure). The perfect matched layer (PML) boundary condition was applied to avoid wave reflections from the system borders. In both cases, results for non-polarized light were obtained as an average of two independent subsimulations, performed for perpendicularly polarized beams (called transverse electric, TE, and transverse magnetic, TM), so that finally:

[1]
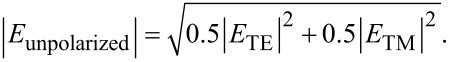


**Figure 13:**
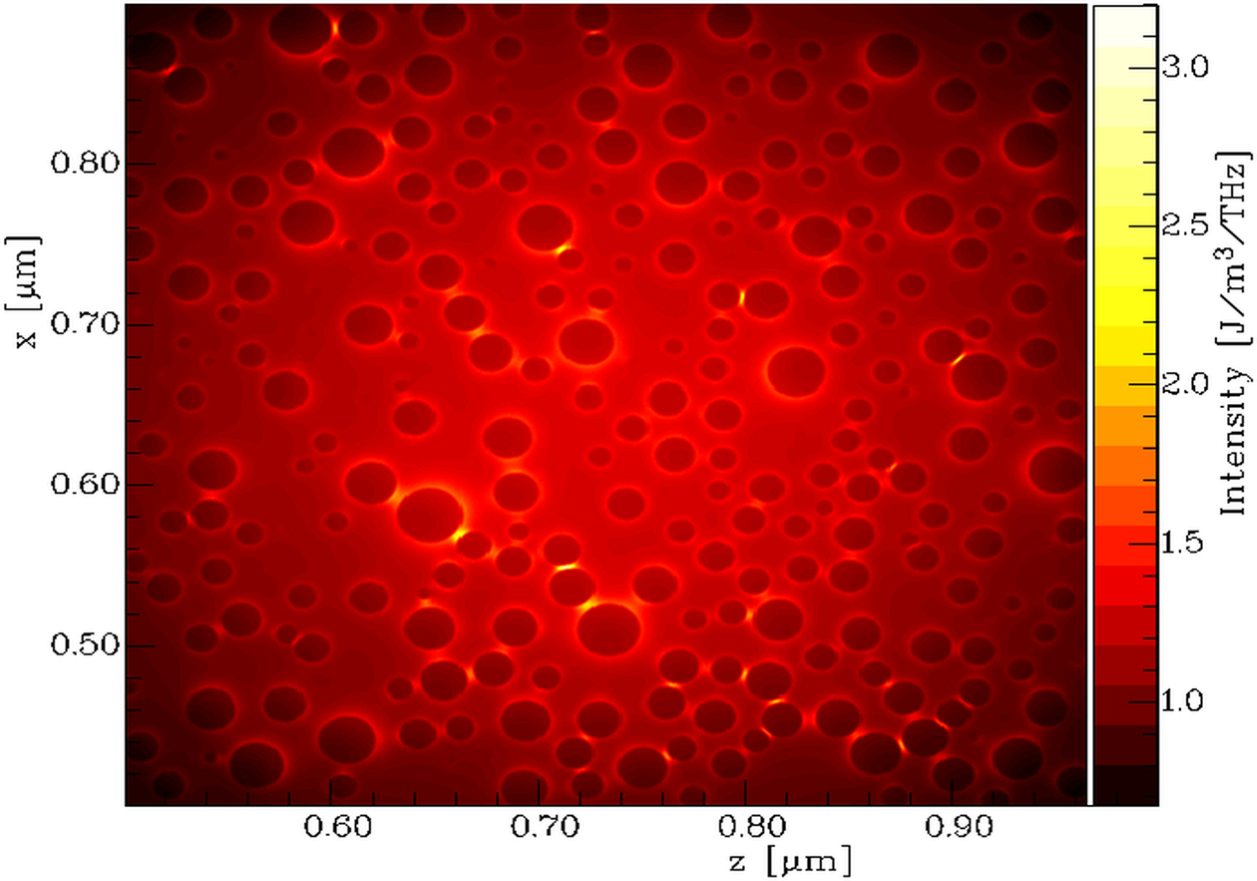
Calculated intensity distribution of the electromagnetic field with unpolarized incident light of a wavelength of 395 nm, integrated 10 nm above the Si surface.

In Figure 13 the intensity distribution of the electromagnetic field in the *zx*-plane, integrated 10 nm above the Si surface is shown. It is the result of the FDTD simulation for 395 nm wavelength. So-called hotspots (places of a strong field enhancement) are clearly visible, especially between nanoparticles located close to each other. The average intensity is generally higher in the center of the sample, because of the intensity profile of the incident light. As a consequence, the surface plasmon resonance was also stronger in that region.

**Figure 14:**
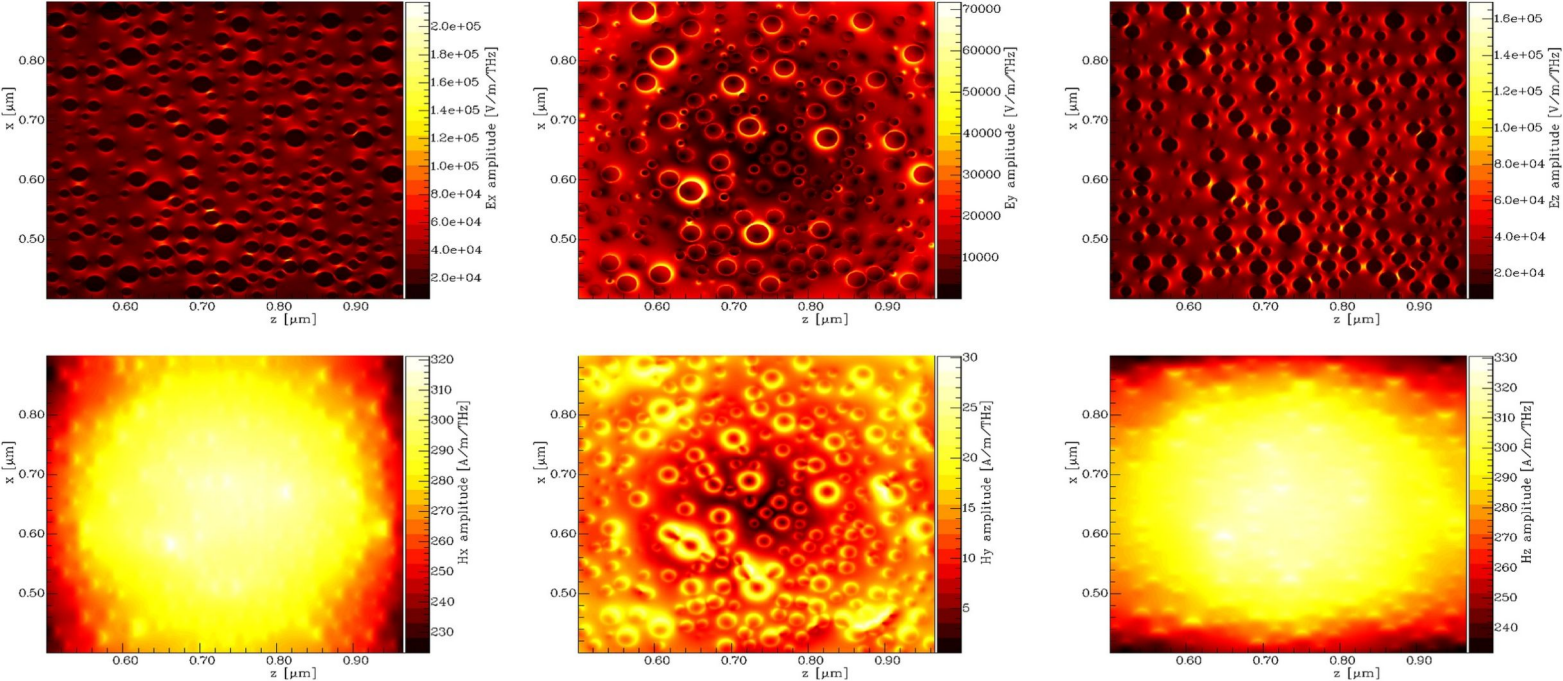
Calculated amplitudes of the components of the electromagnetic field with unpolarized incident light of a wavelength of 395 nm, integrated 10 nm above the Si surface. a) *E_x_* b) *E_y_* c) *E_z_* d) *H_x_* e) *H_y_* f) *H_z_*.

In Figure 14, the amplitudes of particular components of the electromagnetic field are presented as a result of FDTD simulations. Here the amplitudes of the field components parallel to the surface are much stronger than those of the perpendicular *y*-components. This effect is particularly clearly visible for the magnetic field.

**Figure 15:**
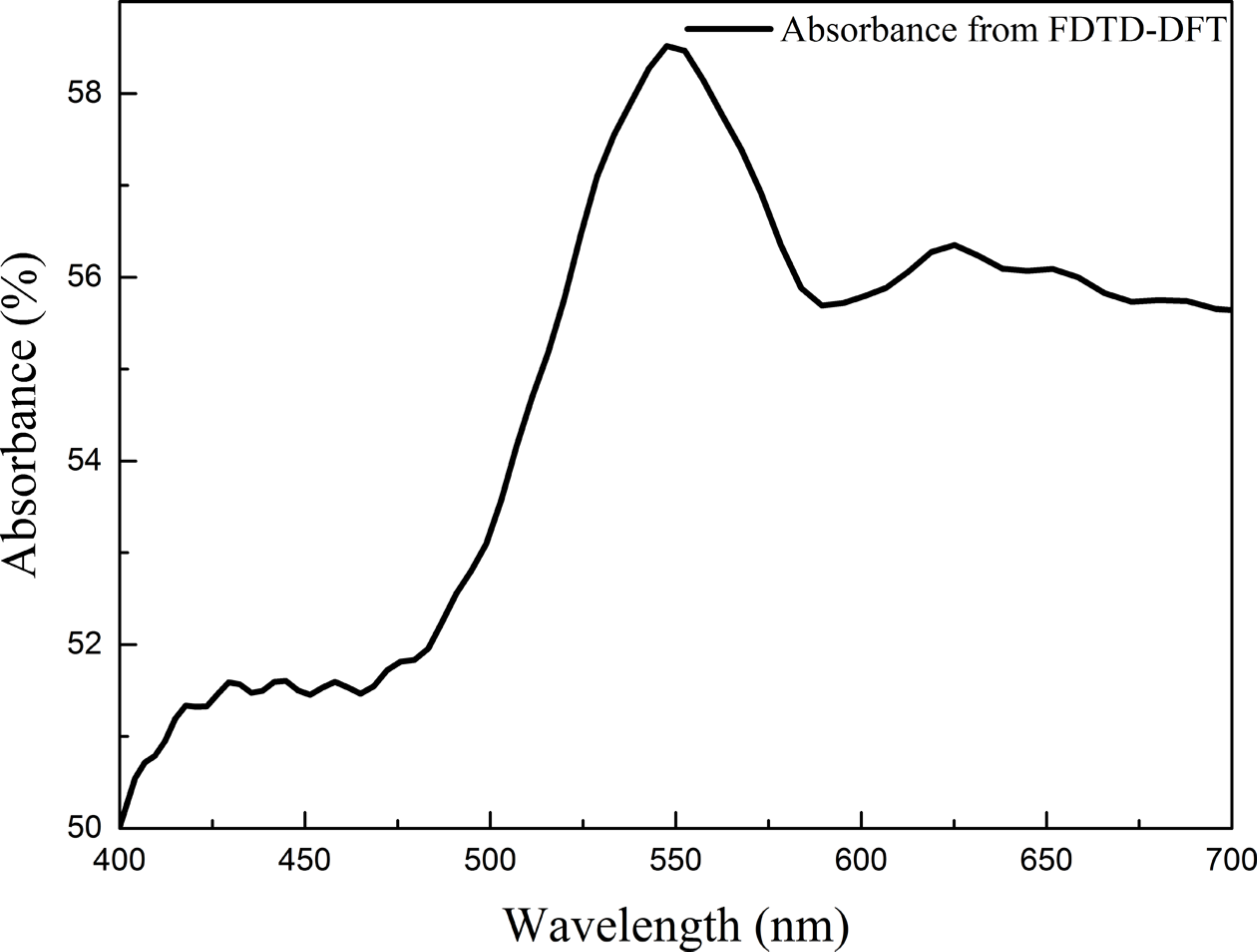
Calculated absorbance (as log(I_o_/I_r_), where I_o_ is an incident flux, and I_r_ – reflected flux), as the function of the incident light wavelength.

The calculated absorbance as a result of the FDTD/DFT simulation is shown in Figure 15. We can observe a quite good agreement with the experimental data presented in Figure 10. It is worth to notice that the exact position of the maximum was reproduced. The remaining discrepancies in the shape of the curve could be explained by the simplified model assuming spherical nanoparticles, and a too coarse grid in the calculations. This is mainly related to software and hardware limitations and may be overcome in a future work.

## Conclusion

Plasmonic platforms based on Au nanostructures have been successfully synthesized from metallic Au thin films deposited on a Si(111) substrate. Two processes should be considered in explaining the formation of nanostructures: dewetting and directional solidification of a eutectic. However, it is not possible to clearly determine what type of dewetting (heterogeneous and spinodal) occurs for the studied samples so our considerations are therefore only qualitative. Simulations of electromagnetic field propagation through the produced samples were performed using FDTD calculations. The calculated absorbance, as a result of FDTD/DFT simulations shows a quite good agreement with experimental data obtained in the UV–vis range. Comparing the obtained experimental results with the computational analysis, it can be concluded that the proposed method of manufacturing nanostructures yields good results. Of course, there are also other methods for the production of nanostructures or metal nanoparticles, for example laser irradiation of thin metallic films deposited on various substrates [29–33]. This method also gives the possibility, by scanning the surface of the sample with a laser or by changing the energy of the pulse or its duration, to create patterns of various sizes of nanostructures. Laser irradiation, however, does not provide the possibility of accurate temperature control, which is extremely important in the case of metals that have a high melting point and form a eutectic compound with the substrate. In addition, the method proposed in our work can be used for the manufacturing of nanostructures on large surfaces, which gives the possibility of application, for example, in plasmonics or photovoltaics. Although, of course, obtaining appropriate homogeneity of nanostructures requires further research.
